# The Opportunity of the Smaller Hospitals

**Published:** 1919-08-16

**Authors:** 


### THE MATRONS' AND SISTERS' DEPARTMENT.

### THE OPPORTUNITY OF THE SMALLER HOSPITALS.

The word "reconstruction" is on every lip; it is applied to every phase of life; it is one of the brightest aspects of the aftermath of war; it lights up a vista of amazing opportunities—opportunities which must be seized now, at once, or they will fade even as the glow fades from the mountain peaks. During the last six months no greater work of reconstruction has taken place than that which has been carried out for the benefit of the nursing profession by the committees of many of our large hospitals. There is a sameness, a conservatism, about hospital life which is difficult to visualise. Each hospital has its individual laws, its sacred traditions, and yet the ethics of institutional life are based on the same principles, they trend in the same direction. The most potent factor in the development of a hospital is the constitution of the committee, for upon the decisions of the committee depend the welfare—nay, the very existence—of the hospital, and consequently the well-being of the staff and the patients entrusted to their charge. The policy of a hospital committee is conservative; it clings to old traditions, and is adverse to modern innovations; and yet within a few months many of our larger hospital committees have passed resolutions which have practically revolutionised the curriculum of their training-schools and broadened to an unprecedented extent the mental outlook of their nursing staffs. They have approached the aspect of reconstruction with an unbiased mind, they have shown a generous desire to understand the aspirations of the present-day probationer, they have provided greater facilities for study, they have increased the salaries of all grades, and they have shortened the hours of the working week.

Unfortunately, many of the smaller provincial hospitals have failed to benefit by the example of their greater brethren. These committees are often composed of laymen grown old in the service of their native town; they regard the hospital as particularly their own, and resent almost as a personal affront any suggestion of change. In pre-war days their training-schools were filled by the over-supply of applicants from the larger hospitals; now, owing to the more extended fields open to women, this surplus no longer exists, and probationers enter the hospitals that have the greater advantages to offer. Yet never had the smaller hospitals a more glorious opportunity, if their committees will but tear the bandage from their eyes and see with a clear mental vision. Let them offer the same training facilities as the larger hospitals; let them prove the worth of the smaller hospitals as teaching centres; in many ways they can offer unique advantages. When the College of Nursing admits members by one-portal examination let the smaller hospitals send up candidates secure in their ability to pass the standard tests, and confident in the teaching they have received. It is not merely a frivolous desire for greater relaxation which draws women to the larger training-schools; it is rather a growing knowledge of the value of scientific teaching, and the desire for a certificate that really counts.

